# Comparison of Upper Airway Ultrasonography Against Quantitative Waveform Capnography for Validating Endotracheal Tube Position in a South Indian Population

**DOI:** 10.7759/cureus.52628

**Published:** 2024-01-20

**Authors:** Swaroop Malamal Pradeep, Honey Ann Benny

**Affiliations:** 1 Department of Emergency Medicine, Valluvanad Hospital Complex Limited, Ottapalam, IND; 2 Department of Emergency Medicine, Jubilee Mission Medical College and Research Institute, Thrissur, IND

**Keywords:** end-tidal carbon dioxide, ultrasonography, quantitative waveform capnography, endotracheal intubation, emergency department

## Abstract

Introduction: The utilization of ultrasonography (USG) is progressively growing to verify the accurate positioning of the endotracheal tube (ETT). Non-detection of the esophageal intubation can be fatal. Various techniques are employed to confirm the placement of the ETT, but none of them are considered optimal. Quantitative waveform capnography (qWC) is often regarded as the most reliable method for this purpose; however, it may not necessarily be accessible and can be expensive. Hence, this investigation was carried out to contrast the use of bedside upper airway USG with qWC in order to confirm the accurate positioning of the ETT following intubation.

Methods: A prospective validation study was undertaken in the emergency department (ED) of Lourdes Hospital, Kochi. This study includes subjects who are of the age group >18 years of either sex requiring intubation in the ED for causes like respiratory failure, cardiac arrest, coma, head injury, and poisoning and cases in which intubation was achieved in the first attempt. The sample size calculated was 77. Intubation in our ED includes both elective and emergency. For all the patients undergoing intubation, consent was taken before the procedure (from close relatives of the patients) by another staff after explaining the procedure to be conducted by the doctor. Following the acquisition of consent, the intubation procedure was executed in accordance with the established hospital protocol. This protocol included verifying the intubation's success as well as employing clinical techniques such as observing bilateral chest expansion, conducting a five-point auscultation, and monitoring pulse oximetry. Furthermore, USG was employed to assess the positioning of the ETT placement. The time taken by each of these methods to confirm tube placement was noted, and the findings were assessed for the sensitivity (SN) and specificity (SP) of USG against the gold standard qWC to confirm endotracheal intubation.

Results: Eighty patients were enrolled in the study. All 80 patients were subjected to both ultrasound and end-tidal carbon dioxide (EtCO_2_). Of the 80 patients, six subjects (7.5%) underwent esophageal intubation, which was observed through the use of USG. Four patients had esophageal intubations and were correctly detected by EtCO_2_. All four esophageal intubations were correctly confirmed by EtCO_2_. Additionally, USG detected six intubations, out of which four were true and two were tracheal which was correctly confirmed by EtCO_2_. The bedside upper airway USG demonstrated an SN of 78 subjects at 97.4% (95% CI: 90.8-99.7%), an SP of 80 subjects at 100% (95% CI: 39.7-100%), a positive predictive value of 80 subjects at 100% (95% CI: 93.8-100%), and a negative predictive value of 53 subjects at 66.7% (95% CI: 33.7-88.7%). A positive test had an infinite likelihood ratio, whereas a negative test had a likelihood ratio of 0.03 (95% CI: 0.01-0.10). The average duration for confirmation by USG was 10.10 seconds.

Conclusion: The study's outcomes highlight the importance of incorporating USG into the clinical toolkit of ED physicians, ultimately contributing to enhanced patient safety and the optimization of endotracheal intubation procedures in the ED.

## Introduction

Mastery of airway techniques is essential for effectively treating severely ill patients upon their admission to the emergency department (ED). Therefore, an ED physician needs to possess proficient airway management abilities, as failure to recognize esophageal intubation might have lethal consequences [1]. This situation may arise during the process of emergency intubation. The incidence rate of esophageal intubation ranges from 6% to 16% [2,3].

Over the years, numerous studies have conducted comparisons of techniques used to differentiate between endotracheal and esophageal tube implantation. In our practice, we utilize numerous techniques, such as inspection during laryngoscopy, expansion of the sternum during breathing, auscultation, capnography, esophageal detection equipment, and chest X-rays. Nevertheless, these approaches exhibit discrepancies in their precision [3-6]. The Advanced Cardiovascular Life Support (ACLS) 2020 Guidelines advocate for the continual utilization of quantitative waveform capnography (qWC) with clinical assessment as a highly dependable approach to validate the precise positioning of an endotracheal tube (ETT) [7]. The study conducted determined that end-tidal carbon dioxide (EtCO_2_) is the most suitable technique for detecting esophageal intubation [7]. A study suggests esophageal detector devices have greater reliability in predicting esophageal intubation in patients with cardiac arrest [8]. Although many approaches are cited to confirm the ETT position, a unique method of verification that is optimal for all situations still does not exist [9-11].

However, two studies are closest to the ideal, which use qWC to validate the ETT position in patients with cardiac arrest post intubation. It documented 100% sensitivity (SN) and 100% specificity (SP) in detecting ETT placement [4,12]. Therefore, qWC is deemed the benchmark for the detection of the position of the ETT. However, qWC has limitations. EtCO_2_ operates by detecting carbon dioxide (CO_2_) in exhaled breath. This can only occur when there is sufficient blood circulation. In situations where there is a decrease in pulmonary blood flow, such as during cardiac arrest or pulmonary embolism, this number exhibits a quick and significant decline [13].

Capnography is readily accessible in operating rooms; however, it is not widely available in many EDs as it is expensive in some countries. Ultrasound is an emerging practice in many EDs, both for the immediate imaging of injuries and for guided medical procedures [14]. Ultrasonographic machines are easy to carry, widely available, and easily reproducible and have good safety records [15]. The purpose of the study was to evaluate and compare the accuracy of upper airway USG and qWC, both of which are considered highly reliable methods, in predicting the correct placement of the ETT during the emergency intubation of critically ill patients.

## Materials and methods

A prospective validation study was undertaken at the Emergency Medicine Department of Lourdes Hospital, Kochi, India, which is a multispecialty National Accreditation Board for Hospitals and Healthcare Providers (NABH)-accredited teaching hospital. The study was conducted between September 2017 and December 2018. The study was done among 80 critically ill adult patients intubated in the ED of Lourdes Hospital in the presence of the primary investigator. The formula used was N=2[Z_alpha+Z_betaxS]²/d², where Z_alpha is 95%, which is the level of significance, Z_beta is 80%, which is the power of the study, d is the clinically significant difference between the two parameters, and SD is the common standard deviation. SN and SP were measured as follows: SN=True Positives/(True Positives+False Negatives), and SP=True Negatives/(True Negatives+False Positives).

Inclusion criteria included patients with an age group >18 years of either sex who were subjected to intubation (elective and urgent) in the ED for causes like respiratory failure, cardiac arrest, coma, head injury, and poisoning and cases in which intubation was achieved in the first attempt (for the purposes of this study, only the first attempt was taken into consideration. It could be either esophageal or tracheal intubation. If the attempt was esophageal, then a reattempt was done, but without performing USG and EtCO_2_). Exclusion criteria included those with anatomical neck distortions due to any cause, such as an enlarged thyroid, abnormalities of the mandible, or maxilla, facial injury, abnormalities of the neck, or burns of the upper airway, or chest. This is because distorted anatomy makes placement of the ultrasound probe difficult, resulting in poor assessment. This study was carried out in Lourdes Hospital after the approval of the Institutional Scientific and Research Committee with l.c. number LH/SC/2017-32. Informed consent (in both English and Malayalam) was obtained from patients and close relatives of comatose or unconscious patients. Ultrasound examination was done in each case by the investigator who underwent a one-month training program in basic ultrasound examination. The primary investigator in our study underwent one month of training in the basics of ultrasound under the guidance of the chief radiologist in our centre, before the onset of the study.

The intubation was conducted by the usual hospital norms, which involve verification using qWC and clinical techniques such as bilateral chest expansion and five-point auscultation. The reference test for esophageal intubation is fall in qWC/absent waveform. Chest radiographs were routinely taken after intubation as part of hospital protocol. USG was employed as a component to determine the positioning of the ETT following the intubation procedure, in conjunction with qWC for all patients involved. We used the static method of confirmation of ETT position, i.e., once intubation was done, the investigator checked the USG to confirm the position. At the same time, it is connected to EtCO_2_ to check the reading time required for both of them to confirm the position; the reading times for both are then compared. The tube was classified as endotracheal if characteristic square qWC was recorded, accompanied by the measurement of CO_2_ levels exceeding 30 mm Hg after six breaths.

Ultrasonography (USG) was performed using a LOGIQ 5 Series ultrasound scanner (GE Medical Systems Co. Ltd., Little Chalfont, UK), which was coupled with a linear transducer operating at a frequency range of 7.5-10 MHz. Following the intubation procedure, the placement of the ETT was promptly confirmed. The probe was positioned horizontally on the front section of the neck, just above the suprasternal notch. The investigator, who had received comprehensive expertise in USG, performed the recommended validation of endotracheal intubation. The tracheal USG was consistently performed by the single investigator in all instances.

The sonographer determined the location of the tube to be in either the trachea or the esophagus using the following method: Tracheal intubation is recommended when just one air-mucosal (A-M) contact is detected, together with reverberation artifacts and posterior shadowing, while esophageal intubation is indicated when two A-M interfaces and posterior shadowing are observed, a condition referred to as the double-tract sign.

**Figure 1:**
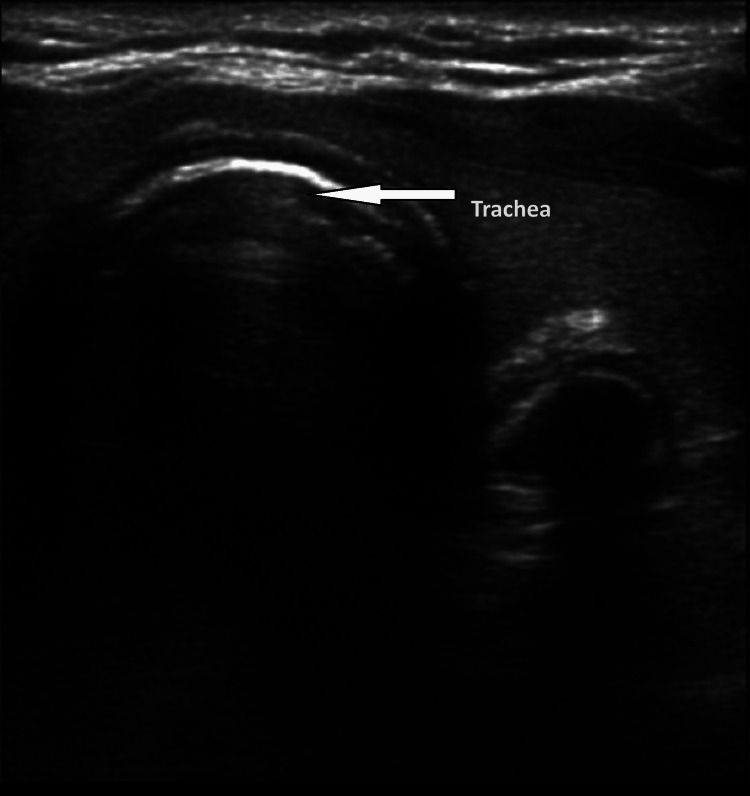
Ultrasonographic image of esophageal intubation showing double-tract sign

**Figure 2:**
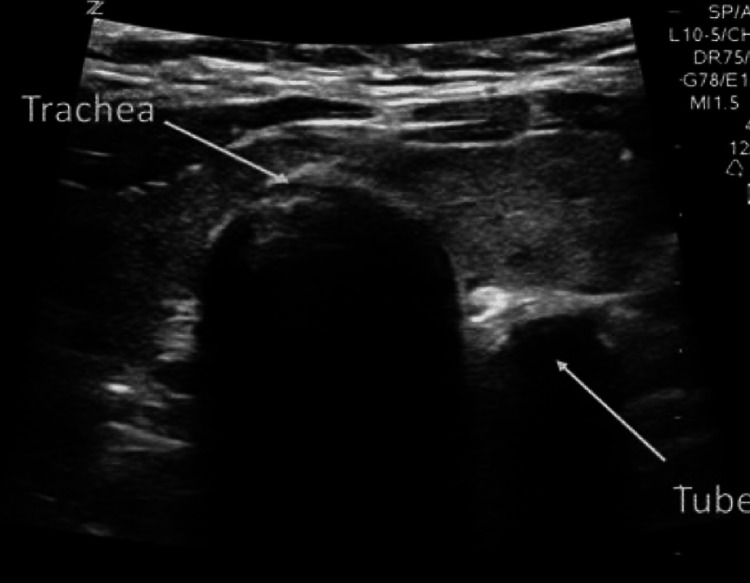
Ultrasonographic image of esophageal intubation

The patient was intubated at time zero, which refers to the specific time period. Subsequently, the lead researcher verified the positioning of the tube and recorded the duration using a timer, with the assistance of the staff nurse. The staff nurse recorded the duration of six cycles and the corresponding quantitative value of CO_2_ in the exhaled air as the capnography time for the capnometer. The staff nurse recorded the duration of time it took for the lead investigator to verify the tube placement using USG, starting from the moment the ultrasound probe was put on the neck (Figure 1 and Figure 2).

The information obtained was entered into a Microsoft Excel sheet (Microsoft Corporation, Redmond, WA), and statistical analysis was performed using IBM SPSS Statistics for Windows, V. 20.0 (IBM Corp., Armonk, NY). Independent t-tests were used to compare normally distributed continuous variables between the two groups. The Mann-Whitney U test was employed for variables that were not normally distributed. Using the chi-squared test, categorical variables between the two groups were compared. A p-value of <0.05 was considered statistically significant. All statistical tests performed were two-tailed. Results were presented as mean, median±SD, counts, and percentages. Confidence intervals (CI) were likely calculated around SN and SP values to provide a range within which the true values were likely to fall, helping quantify the associated uncertainty. Likelihood ratios, including positive and negative likelihood ratios, were calculated to assess the diagnostic accuracy of USG in predicting tracheal or esophageal intubation, offering insights into how much a positive or negative test result changes the odds of the condition being present.

## Results

The age distribution in the study sample was as follows: 61-80 years (52.5%), 41-60 years (31.3%), 80 or more years (11%), and less than 40 years (5%). The mean age was 63.49±14.4 years. Males represented 65% of the 80 patients that were included in the study, and women comprised 35%. Out of the 80 patients, 46.3% were intubated in view of hemodynamic instability, followed by respiratory failure (30%). Around 23.8% underwent intubation to protect the airway (Table 1).

**Table 1:** Demographic data

Variables	Frequency (n)	Percentage (%)
Age group (years)		
Up to 40	4	5%
41-60	25	31.3%
61-80	42	52.5%
Above 80	9	11.3%
Total	80	100%
Gender		
Males	52	65%
Females	28	35%
Indication for intubation		
Hemodynamic instability	37	46.3%
Respiratory failure	24	30%
Airway protection	19	23.8%
Total	80	100%

USG detected tracheal intubation in 92.5% of cases and esophageal intubation in 7.5% of cases. EtCO_2_ detected tracheal intubation in 95% of cases and esophageal intubation in 5% of cases. There were a total of four esophageal intubations. qWC correctly identified four, but USG detected six, out of which two were misinterpreted as esophageal (which was tracheal, maybe because of the short neck) (Table 2).

**Table 2:** Percentage confirmation of esophageal and tracheal intubation USG: ultrasonography; EtCO2: end-tidal carbon dioxide

USG	Frequency (n)	Percentage (%)
Tracheal	74	92.5%
Esophageal	6	7.5%
EtCO_2_		
Tracheal	76	95%
Esophageal	4	5%

The bedside upper airway USG demonstrated an overall accuracy of 97.5% (95% CI: 91.3-99.7%). The bedside upper airway USG demonstrated an SN of 78 subjects at 97.4% (95% CI: 90.8-99.7%), an SP of 80 subjects at 100% (95% CI: 39.7-100%), a positive predictive value (PPV) of 80 subjects at 100% (95% CI: 93.8-100%), and a negative predictive value (NPV) of 53 subjects at 66.7% (95% CI: 33.7-88.7%). A positive test had an infinite likelihood ratio, whereas a negative test had a likelihood ratio of 0.03 (95% CI: 0.01-0.10) (Table 3).

**Table 3:** Sensitivity, specificity, PPV, and NPV of USG for tracheal intubation confirmation CI: confidence interval; PPV: positive predictive value; NPV: negative predictive value; USG: ultrasonography

Variable	Value (n)	95% CI	
		Lower limit	Upper limit
Sensitivity	97.4% (78)	90.8%	99.7%
Specificity	100% (80)	39.7%	100%
PPV	100% (80)	93.8%	100%
NPV	66.7% (53)	33.7%	88.7%
Likelihood ratio (+)	Infinity	-	-
Likelihood ratio (-)	0.03	0.01	0.10
Accuracy	97.5	91.3%	99.7%

The average duration for the validation of ETT by USG was 10.10±2.2 seconds. The minimum duration reported was 6.39 seconds, while the maximum duration was 14.02 seconds. The average duration for the validation of ETT placement using EtCO_2_ was 21.66±3.7 seconds. The minimum recorded time was 14.96 seconds, while the maximum recorded duration was 28.01 seconds (Table 4).

**Table 4:** Mean and SD of duration for confirmation SD: standard deviation; USG: ultrasonography; EtCO2: end-tidal carbon dioxide

Variables	N	Mean±SD	Minimum, maximum	P-value
Duration for confirmation: USG (in seconds)	80	10.10±2.2	6.39, 14.02	0.001
Duration for confirmation: EtCO_2_ (in seconds)	80	21.66±3.7	14.96, 28.01	

Our study also showed that bedside upper airway USG has a very quick mean time of detection of the correct placement of ETT at 10.10 seconds with a standard deviation of 2.2 seconds. This is because the primary investigator in our study underwent one month of training in the basics of ultrasound under the guidance of the chief radiologist in our centre, before the onset of the study. Out of the 80 patients who underwent intubation, 95% were tracheal and 5% were esophageal. Tracheal USG detected tracheal intubation in 92.5% of cases and esophageal intubation in 7.5% of cases. Tracheal USG correctly detected all 5% of esophageal intubations but misinterpreted 2.5% of tracheal intubation as esophageal, probably due to a poor window caused by obesity (short neck). Statistical analysis of the comparison of ultrasound with EtCO_2_ regarding the detection of esophageal intubation gave a p-value of <0.001; that is, ultrasound is more sensitive in detecting esophageal intubation compared to EtCO_2_. The overall accuracy of bedside upper airway USG was 97.5% (95% CI: 91.3-99.7%). The kappa value (Κ) was 0.79, indicating a good agreement between the bedside upper airway USG and qWC. Kappa statistic is used because it gives the strength of agreement over and above that which would have occurred just by chance. Thus, bedside upper airway USG is in concordance with qWC. The SN, SP, PPV, and NPV of the bedside upper airway USG were 97.4% (95% CI: 90.8-99.7%), 100% (95% CI: 39.7-100%), 100% (95% CI: 93.8-100%), and 66.7% (95% CI: 33.7-88.7%). The likelihood ratio of a positive test was infinite, and the likelihood ratio of a negative test was 0.03 (95% CI: 0.01-0.10) (Table 3).

In our study, out of the six esophageal intubations detected by USG, two of them were tracheal which were misinterpreted as esophageal intubations. This is because of the poor window attributed to obesity with short neck. EtCO_2_ correctly identified four esophageal intubations. This means that in obese patients with short necks, EtCO_2_ is more sensitive and specific compared to USG. 

## Discussion

Tracheal USG identified tracheal intubation in 92.5% of cases and esophageal intubation in 7.5% of cases. Tracheal USG correctly identified all 5% of esophageal intubations but erroneously interpreted 2.5% of tracheal intubations as esophageal, probably due to a poor window caused by obesity (short neck).

In 2007, Milling et al. conducted a study that accurately detected all five cases of esophageal intubations out of a total of 40 cases. The SN of the study was 100%, with a 95% CI of 48-100. The USG accurately detected 34 out of 35 tracheal intubations and incorrectly recognized one, disclosing an SP of 97% (95% CI: 90-100) [15]. The study was done by an anesthesiologist in the operating room. Another study by Park et al. in 2009 detected 10% of esophageal intubations (three out of 30) by different ultrasound techniques with an SN, an SP, a PPV, and an NPV for endotracheal intubation of 96.3%, 100%, 100%, and 75%, respectively [16].

Similar results were obtained by Göksu et al. in 2010, which showed an overall SN of 95.7% and an SP of 98.2% [17]. Similarly, a study by Muslu et al. showed an SN of 100% (95% CI: 84-100%) and an SP of 100% (95% CI: 84-100%) [18]. Another study by Chou et al. in December 2013 detected 7.8% (seven out of 89) esophageal intubations [19]. This is similar to our study, in which 7.5% of esophageal intubations were detected by USG. In our study, out of the six esophageal intubations detected by USG, two of them were tracheal, which were misinterpreted as esophageal intubations. 

Studies are comparing real-time (dynamic) and post-intubation (static) assessments. A study by Ma et al. in 2007 suggested that dynamic trans-cricothyroid USG is a precise technique for validating ETT placement during intubation. The dynamic evaluation demonstrated 97% SN and 100% SP for detecting esophageal ETT placement, whereas the post-intubation assessment exhibited 51% SN and 91% SP only [9]. A similar study by Park et al. in 2009 concluded that the use of trans-cricothyroid membrane USG, along with USG lung-sliding evaluation, could be beneficial in verifying ETT intubation in the ED [16]. A study by Abbasi et al. in 2015 showed that the real-time USG was found to have an SN of 98.1% (95% CI: 88.8-99.9%), an SP of 100% (95% CI: 51.6-100%), a PPV of 100% (95% CI: 91.5-100%), and an NPV of 85.7% (95% CI: 42-99.2%) for accurately determining endotracheal intubation [20]. This is similar to our findings, in which SN was 97.4%, SP was 100%, PPV was 100%, and NPV was 66.7%. This is because Abbasi et al. [20] used real-time USG (dynamic), whereas we used immediate post-intubation USG (static).

Three cadaveric studies done in 2006, 2010, and 2015 [17,21,22] showed that esophageal intubations were detected more when the ED physicians were well-trained in USG. Various studies were done to determine the time taken for USG. This study showed that bedside upper airway USG has a very rapid average duration of detection of the correct placement of ETT at 10.10±2.2 seconds. This is because the primary investigator in our study underwent one month of training in the basics of ultrasound under the guidance of the chief radiologist in our centre, before the onset of the study. The detection of ETT placement using the Tracheal Rapid Ultrasound Exam (TRUE) was nine seconds [21]. Our detection time of 10.10 seconds is very close to this value. This again proves that training in ultrasound is essential before performing.

The duration necessary to verify the positioning of an ETT is a crucial consideration for any employed technique. Transtracheal USG can be utilized to confirm the placement of an ETT in real time either during the intubation process or after the intubation is accomplished in a static manner. Compared to post-intubation scanning, real-time sonographic imaging during intubation demonstrated an enhanced SN for detecting esophageal intubation [10,17,21].

Muslu et al. conducted a study in 2011 in which esophageal intubations were detected within three seconds by a trained radiologist using ultrasound. This again emphasizes the need for training before using ultrasound and reinforces the need for ultrasound training for ED physicians [18]. In 2014, Hoffmann et al. found that the absence of sonographic images in the esophagus was completely specific for endotracheal intubation. Additionally, they observed that the presence of a "double-tract sign" was completely sensitive and 91% specific for esophageal intubation. Compared with the intubation, the sonographic diagnosis time was much quicker [23]. This was done by trained ED physicians who used the anterior neck approach with 100% accuracy. In our study, we used the anterior neck approach for USG with 97.5% accuracy with just one month’s training in the basics of USG.

This study's limitations include a relatively small sample size of 80 patients, which may impact the generalizability of the findings to broader populations. Conducted in a single ED at Lourdes Hospital, the study's single-centre nature limits its applicability to different healthcare settings with varying patient demographics and resources. Exclusion criteria, such as anatomical neck distortions and specific medical conditions, introduce potential selection bias, limiting the study's representation of a broader patient population. Moreover, the potential for observer bias in interpreting ultrasound results was not addressed, and operator skill and experience could influence ultrasound accuracy. Additionally, the study lacked long-term follow-up data on patient outcomes and complications related to intubation procedures. Finally, the relatively low NPV of ultrasound suggests its limitations in ruling out esophageal intubation in all cases, emphasizing the need for further research and consideration of these limitations in clinical practice. 

## Conclusions

Both USG and qWC have comparable SN and SP in identifying the tracheal or esophageal position of the ETT. However, USG detected the tube placement faster than qWC. This time difference is statistically significant in a critical situation, and it is of utmost clinical importance. This study also shows that the use of bedside upper airway USG to verify ETT position in a critical situation is reliable and can be very easily and quickly done. A mere one month’s training in the basics of ultrasound helped us to achieve 97.5% accuracy in our study. Hence, even a short spell of training for ED physicians will go a long way in improving the quality of intubation in the ED.
